# Proteomic Analysis of Endometrial Cancer Tissues from Patients with Type 2 Diabetes Mellitus

**DOI:** 10.3390/life12040491

**Published:** 2022-03-28

**Authors:** Muhammad Mujammami, Mohamed Rafiullah, Assim A. Alfadda, Khalid Akkour, Ibrahim O. Alanazi, Afshan Masood, Mohthash Musambil, Hani Alhalal, Maria Arafah, Anas M. Abdel Rahman, Hicham Benabdelkamel

**Affiliations:** 1 University Diabetes Center, King Saud University Medical City, King Saud University, Riyadh 11461, Saudi Arabia; mhmujammami@ksu.edu.sa; 2 Department of Medicine, College of Medicine, King Saud University, Riyadh 11461, Saudi Arabia; aalfadda@ksu.edu.sa; 3 Strategic Center for Diabetes Research, College of Medicine, King Saud University, Riyadh 11461, Saudi Arabia; mrafiullah@ksu.edu.sa; 4 Proteomics Resource Unit, Obesity Research Center, College of Medicine, King Saud University, Riyadh 11461, Saudi Arabia; afsmasood@ksu.edu.sa (A.M.); mthammitone@ksu.edu.sa (M.M.); 5 Obstetrics and Gynecology Department, College of Medicine, King Saud University, Riyadh 11461, Saudi Arabia; kakkour@ksu.edu.sa (K.A.); halhalal@ksu.edu.sa (H.A.); 6 The National Center for Biotechnology (NCB), Life Science and Environment Research Institute, King Abdulaziz City for Science and Technology (KACST), Riyadh 11442, Saudi Arabia; ialenazi@kacst.edu.sa; 7 Department of Pathology, College of Medicine, King Saud University, King Saud University Medical City, Riyadh 11461, Saudi Arabia; mariaarafah@ksu.edu.sa; 8 Metabolomics Section, Center for Genome Medicine, Department of Clinical Genomics, King Faisal Specialist Hospital and Research Centre (KFSHRC), Riyadh 11211, Saudi Arabia; aabdelrahman46@kfshrc.edu.sa; 9 Department of Biochemistry and Molecular Medicine, College of Medicine, Alfaisal University, Riyadh 11533, Saudi Arabia

**Keywords:** uterus, endometrial cancer, diabetes, tissue, proteomics, 2D-DIGE, MALDI-TOF

## Abstract

Endometrial cancer (EC) is the most common form of gynecological cancer. Type 2 diabetes mellitus is associated with an increased risk of EC. Currently, no proteomic studies have investigated the role of diabetes in endometrial cancers from clinical samples. The present study aims to elucidate the molecular link between diabetes and EC using a proteomic approach. Endometrial tissue samples were obtained from age-matched patients (EC Diabetic and EC Non-Diabetic) during surgery. Untargeted proteomic analysis of the endometrial tissues was carried out using a two-dimensional difference in gel electrophoresis (2D-DIGE) coupled with matrix-assisted laser desorption/ionization time-of-flight mass spectrometry (MALDI TOF). A total of 53 proteins were identified, with a significant difference in abundance (analysis of variance (ANOVA) test, *p* ≤ 0.05; fold-change ≥ 1.5) between the two groups, among which 30 were upregulated and 23 downregulated in the EC Diabetic group compared to EC Non-Diabetic. The significantly upregulated proteins included peroxiredoxin-1, vinculin, endoplasmin, annexin A5, calreticulin, and serotransferrin. The significantly downregulated proteins were myosin regulatory light polypeptide 9, Retinol dehydrogenase 12, protein WWC3, intraflagellar transport protein 88 homolog, superoxide dismutase [Cu-Zn], and retinal dehydrogenase 1. The network pathway was related to connective tissue disorder, developmental disorder, and hereditary disorder, with the identified proteins centered around dysregulation of ERK1/2 and F Actin signaling pathways. Cancer-associated protein alterations such as upregulation of peroxiredoxin-1, annexin 5, and iNOS, and downregulation of RDH12, retinaldehyde dehydrogenase 1, SOD1, and MYL 9, were found in the EC tissues of the diabetic group. Differential expression of proteins linked to cancer metastasis, such as the upregulation of vinculin and endoplasmin and downregulation of WWC3 and IFT88, was seen in the patients with diabetes. Calreticulin and alpha-enolase, which might have a role in the interplay between diabetes and EC, need further investigation.

## 1. Introduction

Endometrial cancer (EC) is the most common form of gynecological cancer. It is the sixth most common cancer among women globally [1]. Incidence and mortality rates change widely with regions. It is highest in North America and Northern Europe and lowest in Southeast Asia and Africa [2]. EC comprises 90% of all uterine cancers, and the mean age of women diagnosed with EC is 60, with mostly post-menopausal women being affected [2]. Traditionally, ECs are classified into two types based on their clinical characteristics and estrogen dependency. The estrogen-dependent type I endometrial cancer is associated with endometrial hyperplasia, obesity, and metabolic abnormalities. It comprises 80–90% of all ECs. Type I ECs are usually low-grade with a favorable prognosis. The type II EC is estrogen-independent and is associated with atrophic endometrium [3]. The traditional classification does not account for high histological and genetic heterogeneity. The unopposed circulating estrogen is one of the major risk factors of EC in post-menopausal women. The decrease in progesterone production at menopause shifts the balance towards estrogen. When the circulating estrogen is not counterbalanced sufficiently by progesterone, it causes thickening of the endometrium and increases the risk for endometrial hyperplasia and cancer [4]. The use of hormone replacement therapy without counterbalancing progesterone increased the incidence of endometrial cancers, whereas the addition of progesterone to estrogen mitigated the risk [5]. Studies have identified several other risk factors, such as obesity, diabetes, reproductive factors, medications, diet, and exercise.

Diabetes mellitus is one of the most devastating chronic diseases of modern times, affecting people worldwide. It is associated with an increased risk of certain cancers, including EC [6], where epidemiological data suggest a correlation between diabetes prevalence and the increased incidences of endometrial cancers [7]. A meta-analysis found that women with diabetes have a 72% increased risk of endometrial cancer. Hyperglycemia appears to be associated with a higher risk of endometrial cancer, independent of obesity [8]. High circulating glucose levels may function as the source of precursors of cell proliferation. The EC cells have reportedly increased expression of different glucose transporters [9]. Studies on EC cell lines have shown increased glycolysis and glucose-derived lipogenesis [9]. Antidiabetic drug metformin was found to reverse endometrial hyperplasia, downregulate tumor markers, and improve the survival rate among patients with EC [10].

Chronic hyperglycemia in patients with diabetes provides a conducive environment for proliferative activity. Many in vitro studies and animal experiments have shown the relation between a high-glucose environment and tumor progression [8]. However, the specific etiologic link between type 2 diabetes and endometrial cancer is not entirely understood. Proteomic analysis of endometrial tissues from diabetic rats showed that diabetes promoted endometrial hyperplasia. N-acetylgalactosaminyltransferase 2 (GALNT2) was downregulated in diabetic rats with endometrial hyperplasia. In addition, GALNT2 was lower in the endometrial tissue and blood samples in women with endometrial hyperplasia [11]. To the best of our knowledge, no proteomic studies have investigated the role of diabetes in endometrial cancers from clinical samples. The present study aimed to elucidate the molecular link between diabetes and EC using a proteomic approach. We performed an untargeted proteomic analysis of endometrial cancer tissues obtained from patients with and without diabetes.

## 2. Materials and Methods

### 2.1. Study Design and Patient Selection

Patients attending the outpatient clinics of the Obstetrics and Gynecology-Oncology Department, King Khalid University Hospital, College of Medicine, King Saud University in the age group of 40–75 “years” (age-matched) were recruited for the study. This study included 14 participants (7 EC Diabetic and 7 EC Non-Diabetic). The primary assessment was carried out at clinic appointments. Those patients willing to participate in the study were recruited, and informed consent was taken. The sample size was determined by carrying out a power analysis using the Progenesis SameSpots non-linear dynamics statistical software to determine the minimum number of required biological replicates. The surgeon excised 100 mg of tissue from all 14 patients’ endometrium (7 EC Diabetic and 7 EC Non-Diabetic). Frozen tissue section samples were sent to the pathology department for histopathology examination (Supplementary Table S1). The anthropometric measurements’ biochemical and basic data were also collected. Fresh tissue samples were snap-frozen in liquid nitrogen and kept at −80 °C until they were analyzed.

### 2.2. Tissue Protein Extraction

Tissue samples were thawed and washed 3 times with PBS to remove any blood and other contaminant traces before homogenization. Proteins were extracted from EC tissue samples using a T25 digital ULTRA TURRAX homogenizer (IKA, Königswinter, Germany) directly in lysis buffer (0.5 mL, pH 8.8, 30 mM Tris-HCl, 7 M urea, 2 M thiourea, 2% CHAPS, and a 1× protease inhibitor mix) on ice. The suspension was shaken for 30 min at 4 °C and then sonicated (Microsonicator, Qsonica Sonicators, Newtown, CT, USA; 30% pulse, two intervals of 1 min each, separated by a 1 min gap). Fifty mM dithiothreitol (DTT) was then added and the protein extracts centrifuged (20,000× *g*, 40 min, 4 °C). The contaminants were removed and the supernatants were cleaned by precipitation using a 2D clean-up kit according to the manufacturer’s protocol (GE Healthcare, Uppsala, Sweden) [12].

### 2.3. Protein Labeling with Cyanine Dyes, 2D-DIGE, and Image Scanning

The protein pellets were solubilized in labeling buffer (7 M urea, 2 M thiourea, 30 mM Tris-HCl, 4% CHAPS, pH 8.5). Insoluble material was pelleted by centrifugation (12,000× *g*, room temperature, 5 min); protein concentrations were determined in triplicate using the 2D-Quantkit (GE Healthcare, Illinois, USA). The proteins were labeled with CyDye™ DIGE Fluor minimal dyes (400 pmol/50 μg) (GE Healthcare, Uppsala, Sweden) according to the manufacturer’s recommendations, as previously described by our group [12]. In brief, for each sample, 50 μg of protein was incubated (30 min, on ice, in the dark) with 400 pmol of Cy-3 or Cy-5, freshly dissolved in anhydrous dimethyl formamide (DMF). The reaction was quenched by the addition of lysine (1.0 μL, 10 mM, 10 min, on ice, in the dark). A mixture of an equal amount of all samples was pooled, labeled with Cy2, and used as an internal standard, which was normalized and matched across gels to avoid gel-to-gel variation. A dye-switching strategy was applied during labeling to avoid dye-specific bias (Supplementary Table S2). First-dimension analytical gel electrophoresis was performed, followed by second-dimension sodium dodecyl sulfate-polyacrylamide gel electrophoresis (SDS-PAGE) on 12.5% fixed concentration gels, as previously described [12]. The gels were scanned with Sapphire Biomolecular Imager (Azure Biosystems, Dublin, OH, USA) and digitalized via the image analysis software Sapphire Capture system (Azure Biosystems, Dublin, OH, USA). Preparative gels were prepared using total protein (1 mg) obtained from a pool of equal protein amounts of the 14 samples. As described previously, gels were stained with Colloidal Coomassie Blue [12].

### 2.4. Statistical Analysis

The data for the laboratory values are reported as the mean standard deviation. The statistical significance of the difference between the two groups was determined using an unpaired Student’s t-test, with *p* < 0.05 considered significant. The 2D-DIGE gel pictures were uploaded into the Progenesis SameSpots program (Nonlinear Dynamics, Newcastle upon Tyne, UK) and examined using an automated spot recognition approach for statistical analyses for gel image processing. Although the automated analysis was used to discover all of the spots across all 14 gels, each spot was manually revised and edited for correction if necessary. Log normalized volume was used to quantify differential expression. A cutoff ratio of ANOVA test, *p* ≤ 0.05, and fold difference ≥1.5 was considered significant.

### 2.5. Protein Digestion and MALDI Analysis

As previously described, the Coomassie Blue-stained gel spots were washed and digested [12]. In the end, 0.8 μL from a mixture of tryptic peptides derived from each protein was spotted onto a MALDI target (384 MTP Anchorchip) (800 μm Anchorchip; Bruker Daltonics, Bremen, Germany). The spectra were collected with an UltraflexTerm time-of-flight (TOF) mass spectrometer outfitted with a LIFT-MS/MS device (Bruker Daltonics, Bremen, Germany) at reflector and detector voltages of 21 kV and 17 kV, respectively, as described previously [12]. The PMFs were calibrated against a standard peptide calibration standard II (Bruker Daltonics, Bremen, Germany). The PMFs were assessed using Flex Analysis software (version 2.4, Bruker Daltonics, Bremen, Germany). The MS data were interpreted using BioTools v3.2 (Bruker Daltonics, Bremen, Germany). The peptide masses were searched against the Mascot search algorithm (v2.0.04, updated on 9 May 2021; Matrix Science Ltd, London, UK). The identified proteins were screened for Mascot scores higher than 56 and *p* < 0.05.

### 2.6. Bioinformatics Analysis

The protein interaction networks and functions of tissue proteins differentially expressed in patients with diabetic EC and non-diabetic EC were analyzed using Ingenuity Pathway Analysis (IPA) version 9.0 (Ingenuity Systems, Redwood City, CA, USA). This software aids in determining the functions and pathways most strongly associated with the MS-generated protein list by overlaying the experimental expression data onto networks constructed from published interactions. The identified proteins were additionally classified into different categories according to their function and location using the PANTHER classification system (http://www.pantherdb.org, accessed on 23 January 2022) according to their molecular function, biological process, and location.

## 3. Results

### 3.1. Clinical and Biochemical Data

The demographic and biochemical characteristics of the study patients are presented in Table 1. The mean ages of the study participants in the diabetic and non-diabetic groups were 63.7 ± 10.1 and 58.4 ± 11.4 years, respectively. The body weight and BMI in the diabetic group were significantly higher than in the non-diabetic group. Many of the patients in the diabetic group had severe obesity. HbA1c and blood glucose levels were significantly higher in the diabetic group. All other parameters were similar between the groups.

### 3.2. Proteomic Analysis and Identification of Differentially Expressed Proteins

**Figure 1:**
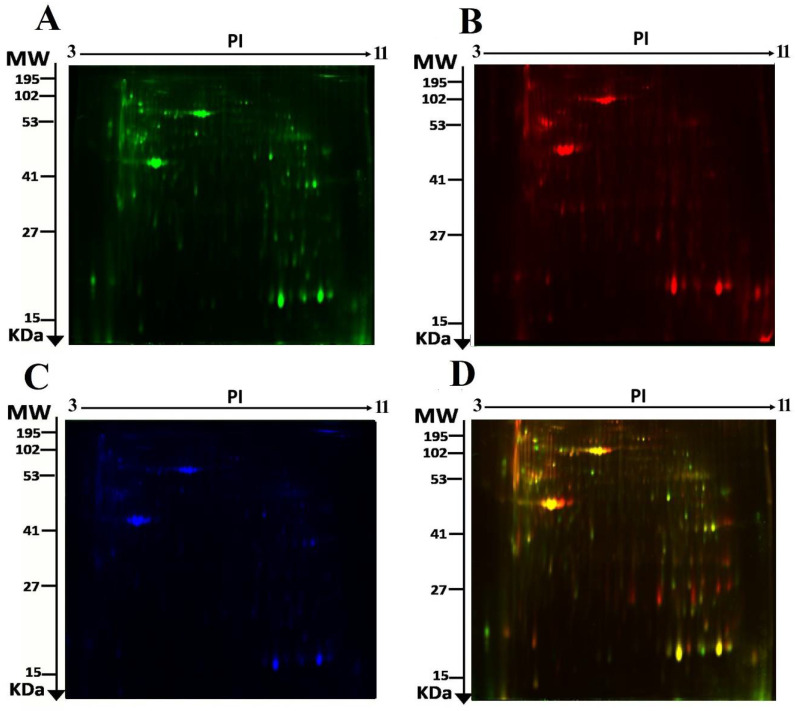
The representative fluorescent protein of a two-dimensional difference in gel electrophoresis (2D-DIGE) containing tissue samples from EC Diabetic samples labeled with Cy3 (**A**), EC Non-Diabetic samples labeled with Cy5 (**B**), pooled internal control labeled with Cy2 (**C**), and merged image (**D**).

We used 2D-DIGE and MALDI-TOF MS to analyze the differential protein expression among 14 endometrial cancer patients (7 EC Diabetic and 7 EC Non-Diabetic, 14 samples from 7 gels). The typical fluorescent protein profiles of a 2D-DIGE of the EC Diabetic samples labeled with Cy3 is shown in Figure 1A, and Figure 1B shows EC Non-Diabetic samples labeled with Cy5; Figure 1C shows a pooled internal control labeled with Cy2, and merged 2D-DIGE gels of samples labeled with Cy3/Cy5 are shown in Figure 1D. 

**Figure 2:**
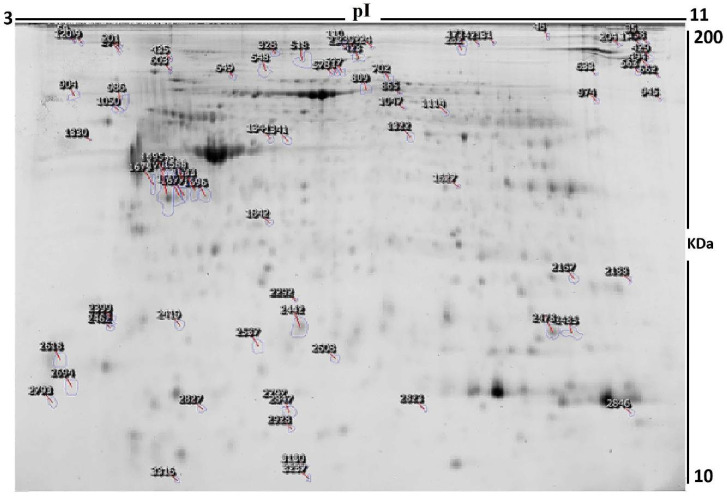
A representative 2D DIGE gel with the numbered spots indicating proteins with differential abundance (defined as fold-change ≥1.5, *p* ≤ 0.05) between EC Diabetic and EC Non-Diabetic samples successfully identified with matrix-assisted laser desorption/ionization-time of flight (MALDI TOF) mass spectrometry (MS). MW, protein molecular weight; pI, isoelectric point.

Figure 2 shows a total of 480 spots matched across all the gels, among which 93 were statistically significantly different (ANOVA, *p* ≤ 0.05; fold-change ≥1.5) between the EC Diabetic and EC Non-Diabetic samples.

The spot patterns were reproducible across the 14 gel images, making the alignment and further analysis possible. An internal standard with Cy2 labeling was used to normalize the complete set of gels and for quantitative differential analysis of the protein levels. Significant changes in protein abundance levels were based on an ANOVA test (i.e., *p* ≤ 0.05 and fold-change ≥1.5). Progenesis statistical software analysis detected 93 protein spots showing a significant increase or decrease in expression between the two states. All differentially abundant protein spots were selected for excision and identification by MALDI-TOF MS.

Peptide mass fingerprints (PMFs) successfully identified 53 out of the 93 protein spots excised from the preparative gel. MALDI-TOF mass spectrometry identified 46 spots as unique protein sequences matched to entries in the SWISS-PROT database by Mascot with high confidence scores (Table 2, Supplementary Table S3). The sequence coverage of the proteins identified by PMF ranged from 9% to 73%. In a few cases, variants of the same protein were found at several locations on the gel (Table 2, Figure 2). Among the 53 proteins identified, 30 protein spots were upregulated, and 23 were downregulated in EC Diabetic compared to EC Non-Diabetic patients. The significantly upregulated proteins included peroxiredoxin-1 (2.87 fold, *p* = 0.018), vinculin (2.86 fold, *p* = 0.032), endoplasmin (2.86 fold, *p* = 0.01), annexin A5 (2.81 fold, *p* = 0.007), calreticulin (2.24 fold, *p* = 0.016), and serotransferrin (2.14 fold, *p* = 0.13). The significantly downregulated proteins included myosin regulatory light polypeptide 9 (−2.47 fold, *p* = 0.006), Retinol dehydrogenase 12 (−2.1 fold, *p* = 0.02), protein WWC3 (−2.1 fold, *p* = 0.03), intraflagellar transport protein 88 homolog (−2.05 fold, *p* = 0.009), superoxide dismutase [Cu-Zn] (−1.92 fold, *p* = 0.02), and retinal dehydrogenase 1 (−1.91 fold, *p* = 0.04); the full list is provided in Table 2. Among the identified proteins, endoplasmin, serotransferrin, vinculin, filamin-A, alpha-actinin-1, heat shock cognate 71 kDa protein and actin, alpha cardiac muscle 1 were found in more than one spot on the gels, which could be associated with their post-translational modifications, cleavage by enzymes, or the presence of different protein species (Table 2).

### 3.3. Principal Component Analysis

**Figure 3:**
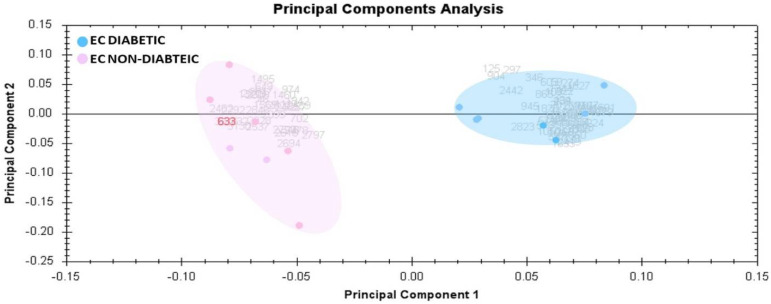
Principal component analysis of the proteomic dataset. Pink dots denote the tissue samples from EC Non-Diabetic and blue dots the EC Diabetic samples. Together, these explained 61.48% of the selected “spot’s” variability values. Colored dots and numbers are the representation of gels and spots, respectively.

Principal component analysis (PCA) on all 53 spot features revealed that the two groups clustered distinctly from one another with a 61.48% cutoff score (Figure 3). The clustering pattern showed that protein intensity changes for selected spots between EC Diabetic and EC Non-Diabetic states were significantly different.

### 3.4. Network Pathway Analysis and Functional Classification of Proteins

**Figure 4:**
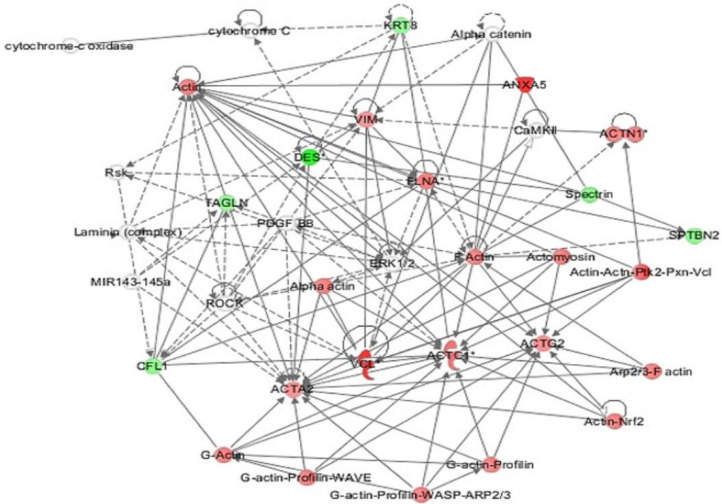
The most enriched interaction network of the differentially expressed proteins in EC Diabetic compared to EC Non-Diabetic states. Red nodes indicate upregulated expression; green nodes indicate downregulated expression. The central nodes of the pathway related to signaling of the ERK1/2 and F Actin were found to be deregulated between the two states. Uncolored nodes are proposed by IPA and indicate potential targets that were functionally coordinated with the differentially expressed proteins. Solid lines indicate direct molecular interactions, and dashed lines represent indirect interactions.

All 53 differentially regulated proteins were subjected to bioinformatic analysis using Ingenuity Pathway Analysis (IPA). The study discovered that 25 proteins interacted directly or indirectly through protein networks (Figure 4). 

**Figure 5:**
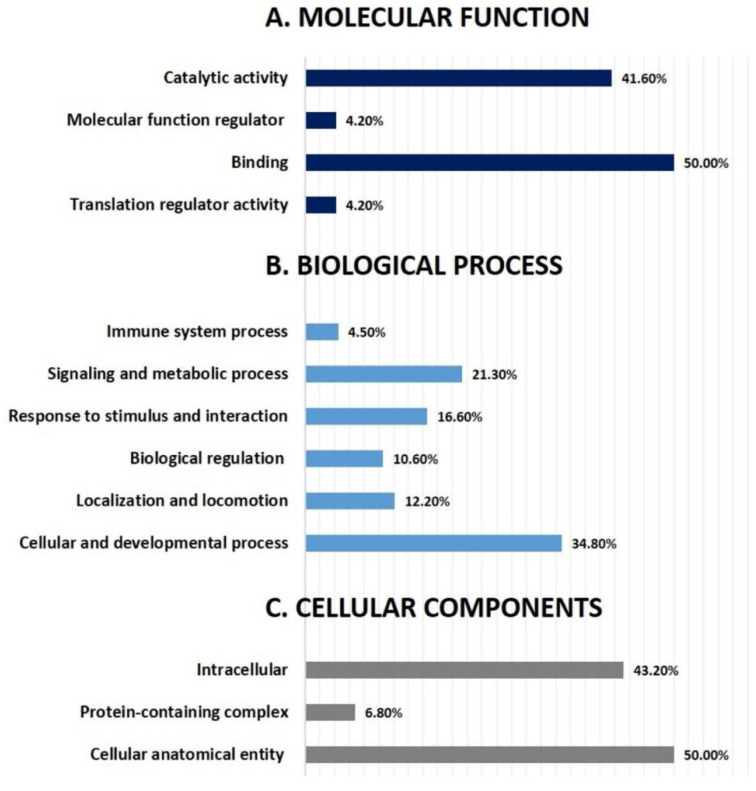
Comparative depiction (%) of identified proteins categorized into groups according to their molecular function (**A**), biological process (**B**), and cellular components (**C**).

The program computes a score based on the best fit obtained from the input data set of proteins and the biological functions database to generate a protein–protein interaction network. The resulting network is enriched for proteins with particular and extensive connections, with interacting proteins represented as nodes and biological links represented as a line. Based on the data, five interaction networks were identified for proteins exhibiting differential expression profiles. The proposed highest-scoring interaction network pathway (score = 28) (Figure 4, Supplementary Figure S1) was related to connective tissue disorder, developmental disorder, and hereditary disorder, with the identified proteins centered around the dysregulation of ERK1/2 and F Actin signaling pathways between the two states. Only the top identified pathway is shown in Figure 4. The canonical pathways enriched in the current dataset are shown in Supplementary Figure S1. The five most interesting enriched canonical pathways included ILK signaling (5.1% overlap, *p* = 4.32 × 10^−12^), Actin cytoskeleton signaling (3.3% overlap, *p* = 2.24 × 10^−8^), Sertoli cell–Sertoli cell junction signaling (3.4% overlap, *p* = 1.40 × 10^−7^), remodeling of epithelial adherence junction (7.4% overlap, *p* = 2.14 × 10^−7^), and dilated cardiomyopathy signaling pathway (4.1% overlap, *p* = 3.87 × 10^−7^). The PANTHER (protein analysis through evolutionary relationships) method was used to classify discovered proteins into molecular activities (Figure 5A), biological processes (Figure 5B), and cellular components (Figure 5C). The functional category showed that most of the differentially expressed proteins identified were enzymes with binding proteins (50%), followed by catalytic activity (42%) (Figure 5A). With regard to biological processes, the majority of the identified proteins were involved in cellular processes and development (35%), followed by cellular and metabolic processes (21%) (Figure 5B). The majority of the identified proteins were located in the cellular, anatomical entity (50%), followed by the intracellular region (43%) (Figure 5C).

## 4. Discussion

The proteomic analysis identified several proteins that are differentially expressed in the endometrial cancer tissues in the presence of diabetes. The bioinformatics analysis indicated dysregulation of the ERK1/2 and F Actin pathways. The ERK1/2 pathway regulates cell proliferation, differentiation, and apoptosis. It is one of the most commonly dysregulated pathways in cancers. Actin polymerization is essential for cancer cell motility and invasion [13]. Dysregulation of the F Actin pathway could indicate the metastatic state of endometrial cancer cells.

### 4.1. Proteins Associated with Cancer

Peroxiredoxin-1, an antioxidant enzyme protecting cells from oxidative stress, was found overexpressed in the EC tissues of the diabetic group in this study. It is suggested to have a proliferative effect and is possibly involved in cancer development and progression [14]. However, peroxiredoxin-1 is also reported to act as a tumor suppressor in some cancers, especially in breast cancers [15]. Upregulation of peroxiredoxin-1 has been reported in endometrial cancer cells. However, the overexpression was not associated with poor prognosis [16]. Even though an antioxidant, peroxiredoxin-1 promotes inflammation by inducing the production of inflammatory cytokines [17]. Annexin A5, used as a probe to detect apoptosis, was highly abundant in the diabetes group. Intracellular annexin 5 is involved in cell membrane repair and calcium channel activity. It inhibits protein kinase C and promotes tumorigenesis and the progression of many cancers. It is also linked to the metastasis and invasion of many cancers [18]. Upregulation of annexin A5 in endometrial cancers has not been reported so far. A previous study reported that annexin 5 was suppressed at the transcription level in endometrial cancer tissue [19]. In cervical cancers, annexin A5 was higher at the protein level and suppressed at mRNA level [19]. Inducible nitric oxide synthase (iNOS) promotes angiogenesis in cancer cells. Our results show that iNOS was overexpressed in the diabetic group. iNOS plays a significant role in the growth and maintenance of tumor cells by promoting angiogenesis. Overexpression of iNOS in endometrial tissue was associated with increasing grades of EC [20]. It increased microvascular density and deep myometrial invasion in endometrial cancer tissues [21].

Retinoic acid is known to suppress the estrogen-induced proliferation in endometrial hyperplasia and metaplasia [22]. Retinol dehydrogenase 12 (RDH 12), an enzyme that converts retinol to retinaldehyde during retinoic acid synthesis, is usually expressed in endometrial stromal tissues [23]. It was significantly downregulated in the endometrial tissues of diabetes patients in our study. Retinoic acid suppresses tumorigenesis by inhibiting cell proliferation and angiogenesis. Human endometrial cancer cells express retinoic acid receptors, and its activation inhibits tumor growth [24]. The downregulation of RDH 12 is likely to decrease retinoic acid levels in endometrial cells. Another enzyme involved in retinoic acid synthesis, retinal dehydrogenase 1, was also found lower in abundance in the diabetic group. This shows a substantial downregulation of retinoic acid synthesis in the endometrial cancer cells of patients with diabetes. Type 2 diabetes is reported to be associated with lower levels of vitamin A [25], and obesity is linked with lower levels of retinoic acid through the downregulation of RDH and/or upregulation of retinal dehydrogenase [26]. Animal studies showed that retinal dehydrogenase 1-deficient mice were more insulin-sensitive and tolerant [27]. In our study, patients in the diabetic group exhibited severe obesity (Table 1).

Therefore, the downregulation of RDH12 in the present study is as expected, but the downregulation of retinal dehydrogenase in the diabetic group appears contradictory. The downregulation of RDH12 decreases the production of retinaldehyde, a substrate of retinal dehydrogenase 1. With less substrate, there would have been less requirement for the enzyme, which might have led to the downregulation of retinal dehydrogenase 1. Since the overexpression of aldehyde dehydrogenase isoforms was associated with enhanced proliferation and poor prognosis [28], the downregulation may be expected to be beneficial. However, downregulation of these enzymes would also decrease retinoic acid synthesis, a suppressor of proliferation and angiogenesis [24]. In the present study, the overall effect of downregulation RDH12 and retinal dehydrogenase 1 in patients with diabetes appears to be associated with a poor prognosis of EC as more patients in the diabetic group had a high-grade stage 3 EC (Supplementary Table S1).

Superoxide dismutase [Cu-Zn] (SOD1) is an antioxidant enzyme that removes the reactive oxygen species. Its production is increased under oxidative stress conditions. In EC tissues, the protein level of SOD1 was found downregulated in all stages [29]. Our results show that the SOD1 expression in the endometrial tissues of the diabetic group was significantly lower. Myosins are actin-dependent molecules that generate force by utilizing ATP. Myosin regulatory light polypeptide 9 (MYL 9), a motor protein from the myosin superfamily, was found significantly downregulated in the diabetic group. The expression of MYL 9 in cancer cells differs with different types of cancers. In ECs, the expression of MYL 9 has been reported to be lower than in the normal tissues [30]. As the proteins associated with cancer cells are more expressed in patients with diabetes, it reflects the aggressive nature of cancer in these patients.

### 4.2. Proteins Associated with Cancer Metastasis

In this study, vinculin, an actin-binding protein, was found highly expressed in the endometrial cancer tissues of patients with diabetes. It is usually localized in focal adhesions and cell-adherence junctions. As a mediator of force transfer between the extracellular matrix and cytoskeleton, vinculin promotes tumor cell invasiveness [31]. Endoplasmin is another protein associated with cancer metastasis, and was highly expressed in the endometrial cancer tissue of patients with diabetes. It is a heat shock protein beta family member. Upregulation of endoplasmin in cancer tissues was associated with poor prognosis and survival [32]. WWC family proteins are cytosolic scaffolding proteins that regulate cell proliferation and invasion via the Hippo signaling pathway. Protein WWC3 was found downregulated in the present study. Lower expression of protein WWC3 was reported in lung cancer cells. It was associated with weak cell differentiation, metastasis, poor prognosis, and a low survival rate in patients with lung cancer [33]. Knockdown of WWC3 enhanced the epithelial–mesenchymal transition of lung cancer cells [34]. It has not been reported in EC so far. Downregulation of WWC3 in diabetic groups might indicate the aggressive nature of EC in the presence of diabetes. Intraflagellar transport protein 88 homolog (IFT88), also known as TG737, is a protein known to suppress the invasion and migration of cancer cells when overexpressed [35]. Silencing IFT88 resulted in the increased proliferation, migration, and invasion of hepatocellular carcinoma [36]. It was found lower in abundance in patients with diabetes and EC in the current study. Several proteins associated with cancer invasiveness and metastasis were linked to the diabetic group in this study.

### 4.3. Proteins with the Possible Interplay between Diabetes and EC

Calreticulin was found upregulated in the endometrial cancer tissues of the diabetic group. It is involved in calcium homeostasis and glycoprotein folding. Overexpression of calreticulin was reported to be associated with longer survival in endometrial cancer patients [37]. It was found to be higher in the early stage and decreased in the advanced stages of EC [29]. The clinical characteristics of the patients in our study show that more patients had a higher grade of EC in the diabetic group than the control group. On the other hand, calreticulin is an indicator of stress in the endoplasmic reticulum, which is linked with the pathophysiology of diabetes. The calreticulin level was found to be higher in obese and insulin-resistant individuals [38]. It appears that the preexisting diabetic conditions might have augmented the overexpression of calreticulin. Alpha-enolase is a glycolytic enzyme expressed ubiquitously in most tissues. It is reported to be upregulated in EC tissues, and its overexpression was associated with an unfavorable prognosis in patients with EC [39]. In another study, proteomic analysis of EC tissues revealed the upregulation of alpha-enolase in the higher-stage ECs compared to healthy tissues [29]. However, our study results show that alpha-enolase levels were lower in the EC tissue of patients with diabetes. We speculate a possible interference from the underlying diabetic conditions in the expression of alpha-enolase protein in the EC tissue. A study on db/db diabetic mice demonstrated that inhibiting the non-glycolytic functions of alpha-enolase produced an antidiabetic effect [40]. Therefore, the possible effect of antidiabetic treatment in downregulating the alpha-enolase enzyme needs to be explored further.

The results demonstrate that the proteins favoring the proliferation, angiogenesis, invasiveness, and metastasis of cancers are significantly expressed in the EC tissues of patients with diabetes. In addition, the EC tissues from the diabetic group showed altered expression of proteins that are usually associated with cancers, indicating the severity of cancer in this group, which is further explained by the presence of more patients with high-grade and higher-stage EC in the diabetic group. Therefore, it is not clear whether the severity of the EC reflected in the altered protein expression pattern is linked to diabetes. The possible effect of diabetes or antidiabetic treatment on the upregulation of calreticulin and downregulation of enolase needs further investigation. A major limitation of this study is not having a matched control group for the EC stage and grades. Protein expression might have differed between different categories of EC stages. We also did not include a normal control group. Comparison with a healthy group would have revealed whether the expression seen in the diabetes group is anomalous or not.

## 5. Conclusions

The present study revealed EC tissues’ differential proteomic expression pattern in patients with and without diabetes. Alterations in the proteins that are usually associated with cancer, including the upregulation of peroxiredoxin-1, annexin 5, and iNOS, and down-regulation of RDH12, retinaldehyde dehydrogenase 1, SOD1, and MYL 9, were found in the EC tissues of the diabetic group. In addition, protein changes associated with cancer metastasis, such as the upregulation of vinculin and endoplasmin, and downregulation of WWC3 and IFT88, were seen in the patients with diabetes. Proteins associated with poor prognosis of cancer were significantly expressed in the diabetic group, except calreticulin and alpha-enolase. The altered expression of these two proteins in the EC tissues may possibly be due to the underlying diabetic conditions. The bioinformatics analysis indicated that the ERK1/2 and F Actin pathways were dysregulated, and these pathways are linked with cell proliferation, differentiation, invasiveness, and apoptosis.

## Figures and Tables

**Table 1:** Clinical and biochemical characteristics of the study population.

Parameters	EC Diabetic (n = 7) Mean ± SD	EC Non-Diabetic (n = 7) Mean ± SD	*p*-Value
**Age** (years)	63.7 ± 10.1	58.4 ± 11.4	0.375
**Height** (cm)	151.8 ± 1.3	151.6 ± 1.5	1.0000
**Weight** (kg)	96.8 ± 10.3	67.1 ±12.9	0.0005 *
**BMI** (kg/m^2)^	42.0 ± 4.9	29.1 ± 5.3	0.0005 *
**HbA1C** (%)	7.0 ± 0.2	6.0 ± 0.3	<0.0001 *
**Total cholesterol** (mmol/L)	4.2 ± 1.0	3.7 ± 1.6	0.496
**LDL** (mmol/L)	2.5 ± 1.0	2.1 ± 1.5	0.496
**HDL** (mmol/L)	1.1± 0.5	1.2 ± 0.2	0.632
**TG** (mmol/L)	1.4 ± 0.7	0.7 ± 0.6	0.110
**Urea** (mmol/L)	4.8 ± 2.0	4.3 ± 1.5	0.606
**Creatinine** (µmol/L)	56.0 ± 9.3	52.6 ± 9.5	0.511
**Glucose** (mmol/l)	10.5 ± 5.5	5.4 ± 0.5	0.031

BMI: Body mass index; HbA1C: Hemoglobin A1c; HDL: High-density lipoprotein; LDL: Low-density lipoprotein; TG: Triglyceride; * statistically significant.

**Table 2:** Proteins identified with changes in abundance between EC Diabetic (ECD) and EC Non-Diabetic (ECND) tissue samples. Values for the average ratio between the two states are shown with their corresponding levels of fold-changes and one-way ANOVA (*p* < 0.05) using 2D-DIGE. Analysis type: MALDI-TOF; database: SwissProt; taxonomy: Homo sapiens.

Sl No.	Spot No. ^a^	Accession No.	Protein Name	MASCOT ID	*p*-Value ^b^ (ANOVA)	Ratio ECD/ECND ^c^	Exp ^d^
1	633	Q96LW4	DNA-directed primase/polymerase protein	PRIPO_HUMAN	8.29 × 10^−4^	−1.5	DOWN
2	274	P14625	Endoplasmin	ENPL_HUMAN	0.002	2.44	UP
3	2462	P24844	Myosin regulatory light polypeptide 9	MYL9_HUMAN	0.01	−2.47	DOWN
4	1627	P04406	Glyceraldehyde-3-phosphate dehydrogenase	G3P_HUMAN	0.01	1.78	UP
5	1691	P08758	Annexin A5	ANXA5_HUMAN	0.01	2.81	UP
6	3130	Q13099	Intraflagellar transport protein 88 homolog	IFT88_HUMAN	0.01	−2.05	DOWN
7	109	P14625	Endoplasmin	ENPL_HUMAN	0.01	2.86	UP
8	2608	P60174	Triosephosphate isomerase	TPIS_HUMAN	0.01	−1.77	DOWN
9	282	P02787	Serotransferrin	TRFE_HUMAN	0.01	2.14	UP
10	986	P27797	Calreticulin	CALR_HUMAN	0.02	2.24	UP
11	131	P13639	Elongation factor 2	EF2_HUMAN	0.02	1.78	UP
12	2167	Q06830	Peroxiredoxin-1	PRDX1_HUMAN	0.02	2.87	UP
13	46	O15020	Spectrin beta chain, non-erythrocytic 2	SPTN2_HUMAN	0.02	−1.6	DOWN
14	2537	P02766	Transthyretin	TTHY_HUMAN	0.02	−1.5	DOWN
15	1322	P06733	Alpha-enolase	ENOA_HUMAN	0.02	−1.9	DOWN
16	2694	P00441	Superoxide dismutase [Cu-Zn]	SODC_HUMAN	0.02	−1.92	DOWN
17	518	P18206	Vinculin	VINC_HUMAN	0.02	1.79	UP
18	172	P21333	Filamin-A	FLNA_HUMAN	0.02	1.67	UP
19	548	P12814	Alpha-actinin-1	ACTN1_HUMAN	0.02	−1.5	DOWN
20	2485	P23528	Cofilin-1	COF1_HUMAN	0.03	−1.6	DOWN
21	2823	P05413	Fatty acid-binding protein, heart	FABPH_HUMAN	0.03	1.77	UP
22	110	P21333	Filamin-A	FLNA_HUMAN	0.03	1.79	UP
23	234	P12110	Collagen alpha-2(VI) chain	CO6A2_HUMAN	0.03	1.79	UP
24	224	P18206	Vinculin	VINC_HUMAN	0.03	2.86	UP
25	2687	Q96NR8	Retinol dehydrogenase 12	RDH12_HUMAN	0.02	−2.1	DOWN
26	346	Q9HAE3	EF-hand calcium-binding domain-containing protein 1	EFCB1_HUMAN	0.03	−1.8	DOWN
27	2319	Q9ULE0	Protein WWC3	WWC3_HUMAN	0.04	−2.1	DOWN
28	341	P11142	Heat shock cognate 71 kDa protein	HSP7C_HUMAN	0.04	1.9	UP
29	307	P12814	Alpha-actinin-1	ACTN1_HUMAN	0.04	1.7	UP
30	1860	P62736	Actin, aortic smooth muscle	ACTA_HUMAN	0.09	1.5	UP
31	603	Q92737	Ras-like protein family member 10A	RSLAA_HUMAN	0.04	2.1	UP
32	1569	Q96S15	GATOR complex protein WDR24	WDR24_HUMAN	0.04	1.7	UP
33	2827	Q9NQ76	Matrix extracellular phosphoglycoprotein	MEPE_HUMAN	0.05	−1.6	DOWN
34	2846	Q5VWT5	FYN-binding protein 2	FYB2_HUMAN	0.05	−1.56	DOWN
35	1114	P00352	Retinal dehydrogenase 1	AL1A1_HUMAN	0.05	−1.91	DOWN
36	2559	Q01995	Transgelin	TAGL_HUMAN	0.05	−1.5	DOWN
37	2928	P06702	Protein S100-A9	S10A9_HUMAN	0.05	−1.5	DOWN
38	1842	P35232	Prohibitin	PHB_HUMAN	0.05	−1.91	DOWN
39	1050	P08670	Vimentin	VIME_HUMAN	0.05	1.5	UP
40	2399	P35228	Nitric oxide synthase, inducible	NOS2_HUMAN	0.01	2.02	UP
41	865	P02545	Prelamin-A/C	LMNA_HUMAN	0.01	−1.82	DOWN
42	702	P02787	Serotransferrin	TRFE_HUMAN	0.01	1.94	UP
43	2847	Q9BWT1	Cell division cycle-associated protein 7	CDCA7_HUMAN	0.01	−1.5	DOWN
44	1344	P05787	Keratin, type II cytoskeletal 8	K2C8_HUMAN	0.01	−1.5	DOWN
45	1341	P68032	Actin, alpha cardiac muscle 1	ACTC_HUMAN	0.01	1.96	UP
46	571	Q9C0H9	SRC kinase signaling inhibitor 1	SRCN1_HUMAN	0.01	−1.5	DOWN
47	1696	P68032	Actin, alpha cardiac muscle 1	ACTC_HUMAN	0.01	1.5	UP
48	2478	P63267	Actin, gamma-enteric smooth muscle	ACTH_HUMAN	0.01	1.56	UP
49	1633	P62937	Peptidyl-prolyl cis-trans isomerase A	PPIA_HUMAN	0.01	−1.61	DOWN
50	2442	O95789	Zinc finger MYM-type protein 6	ZMYM6_HUMAN	0.01	1.55	UP
51	239	Q8IYX0	Zinc finger protein 679	ZN679_HUMAN	0.01	1.59	UP
52	1588	P17661	Desmin	DESM_HUMAN	0.01	2.17	UP
53	649	P11142	Heat shock cognate 71 kDa protein	HSP7C_HUMAN	0.01	1.55	UP

^a^ Protein accession number for SWISSPROT Database. ^b^ *p*-Value (ANOVA). ^c^ Ratio between the groups. ^d^ Protein expression between the groups.

## Supplementary Materials

The following are available online at https://www.mdpi.com/article/10.3390/life12040491/s1: Figure S1: Canonical pathways; Table S1: Cancer stage characteristics; Table S2: Experimental design; Table S3: Mass spectrometry list of significant differentially abundant proteins.
