# Supplementary material for: Proteomic Analysis of Endometrial Cancer Tissues from Patients with Type 2 Diabetes Mellitus
Source: Life (Basel). 2022 Mar 28;12(4):491. doi: 10.3390/life12040491 (PMC9030544; doi:10.3390/life12040491)
Supplement: Supplementary file 1 [file life-12-00491-s001.zip › life-1647811-supplementary.pdf]

life

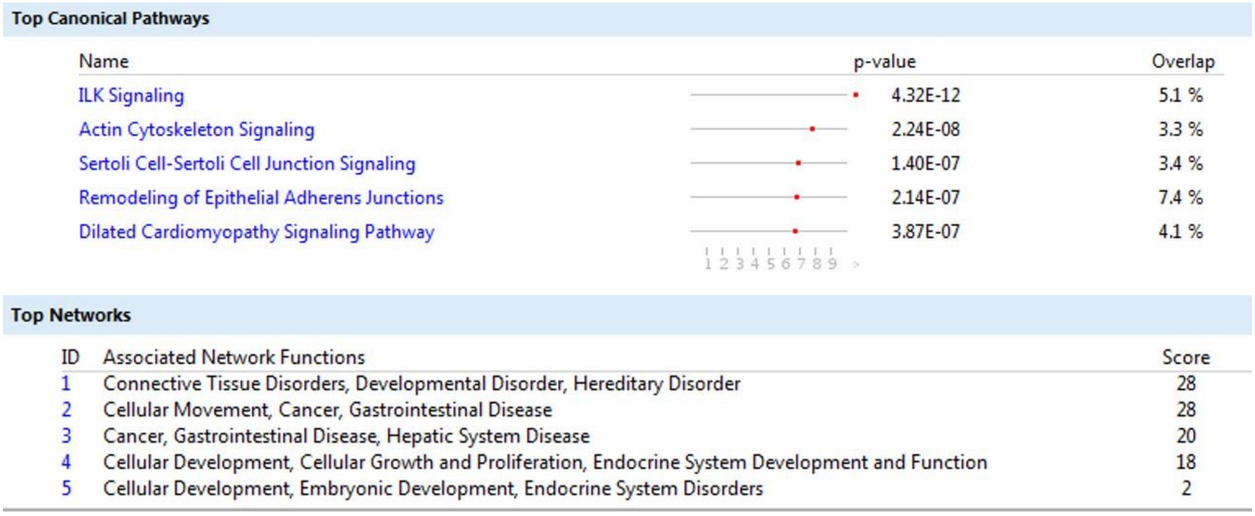

Top Canonical Pathways

Name		p-value	Overlap
ILK Signaling		4.32E-12	5.1 %
Actin Cytoskeleton Signaling		2.24E-08	3.3 %
Sertoli Cell-Sertoli Cell Junction Signaling		1.40E-07	3.4 %
Remodeling of Epithelial Adherens Junctions		2.14E-07	7.4 %
Dilated Cardiomyopathy Signaling Pathway		3.87E-07	4.1 %

Top Networks

ID	Associated Network Functions	Score
1	Connective Tissue Disorders, Developmental Disorder, Hereditary Disorder	28
2	Cellular Movement, Cancer, Gastrointestinal Disease	28
3	Cancer, Gastrointestinal Disease, Hepatic System Disease	20
4	Cellular Development, Cellular Growth and Proliferation, Endocrine System Development and Function	18
5	Cellular Development, Embryonic Development, Endocrine System Disorders	2

Figure S1. Pathways and canonical pathways identified in the IPA functional analysis.

Table S1. Cancer stage characteristics of the study population.

Gel	Sample Id	Diagnosis	Stage/Grade
1	EC-D1	Endometrioid Endometrial Adenocarcinoma	Stage 1A/G2
2	EC-D2	Endometrioid Endometrial Adenocarcinoma	Stage 1A/G2
3	EC-D3	Endometrioid Endometrial Adenocarcinoma	Stage 3A/G3
4	EC-D4	Endometrioid Endometrial Adenocarcinoma	Stage 3A/G3
5	EC-D5	Endometrioid Endometrial Adenocarcinoma	Stage 1A/G2
6	EC-D6	Endometrioid Endometrial Adenocarcinoma	Stage 1A/G2
7	EC-D7	Endometrioid Endometrial Adenocarcinoma	Stage 3A/G3
8	EC-ND1	Endometrioid Endometrial Adenocarcinoma	Stage 1A/G3
9	EC-ND2	Endometrioid Endometrial Adenocarcinoma	Stage 1A/G3
10	EC-ND3	Endometrioid Endometrial Adenocarcinoma	Stage 1AG3
11	EC-ND4	Endometrioid Endometrial Adenocarcinoma	Stage 1B/G3
12	EC-ND5	Endometrioid Endometrial Adenocarcinoma	Stage 1B/G3
13	EC-ND6	Endometrioid Endometrial Adenocarcinoma	Stage 1/G2
14	EC-ND7	Endometrioid Endometrial Adenocarcinoma	Stage 1/G2

EC-D: Endometrial Cancer Diabetic group, EC-ND: Endometrial Cancer Non-diabetic group

Table S2. Experimental design: 14 samples run on 7 2D-PAGE gels, samples were labeled randomly with Cy3 and Cy5, and a pooled sample was used as an internal standard and was stained with Cy2.

Gel	Cy3	Cy5	Cy2
**1**	**EC-D1**	**EC-ND1**	**Pooled sample**
**2**	**EC-ND2**	**EC-D2**	**Pooled sample**
**3**	**EC-D3**	**EC-ND3**	**Pooled sample**
**4**	**EC-ND4**	**EC-D4**	**Pooled sample**
**5**	**EC-D5**	**EC-ND5**	**Pooled sample**
**6**	**EC-ND6**	**EC-D6**	**Pooled sample**
**7**	**EC-D7**	**EC-ND7**	**Pooled sample**

EC-D: ENDOMETRIAL CANCER DIABETIC; EC-ND: ENDOMETRIAL CANCER NONDIABETIC.

Table S3. Mass spectrometry list of significant differentially abundant proteins between EC. Diabetic (ECD) and EC Non-Diabetic (ECND) identified in endometrial tissue samples, using 2DDIGE-MALDI-TOF. Protein name, accession number, Mascot score, MS % coverage, protein MW and pI values according to Uniprot database are listed.

Sl no:	Spot No[a]	Accession No[b]	Protein Name	MASCOT ID	Pi[c]	MW[d]	Cov%	Score[e]

1	633	Q96LW4	DNA-directed primase/polymerase protein	PRIPO_HUMAN	5.19	65169	22	57
2	274	P14625	Endoplasmin	ENPL_HUMAN	4.76	92696	14	58
3	2462	P24844	Myosin regulatory light polypeptide 9	MYL9_HUMAN	4.80	19871	66	81
4	1627	P04406	Glyceraldehyde-3phosphate dehydrogenase	G3P_HUMAN	8.57	36201	50	117
5	1691	P08758	Annexin A5	ANXA5_HUMAN	4.94	35971	49	143
6	3130	Q13099	Intraflagellar transport protein 88 homolog	IFT88_HUMAN	6.20	94781	28	57
7	109	P14625	Endoplasmin	ENPL_HUMAN	4.76	92696	25	127
8	2608	P60174	Triosephosphate isomerase	TPIS_HUMAN	6.45	26938	40	77
9	282	P02787	Serotransferrin	TRFE_HUMAN	6.81	79280	28	57
10	986	P27797	Calreticulin	CALR_HUMAN	4.29	48283	21	57
11	131	P13639	Elongation factor 2	EF2_HUMAN	6.41	96246	17	60
12	2167	Q06830	Peroxiredoxin-1	PRDX1_HUMAN	8.27	22324	69	161
13	46	O15020	Spectrin beta chain, non-erythrocytic 2	SPTN2_HUMAN	5.79	272496	18	57
14	2537	P02766	Transthyretin	TTHY_HUMAN	5.52	15991	73	89
15	1322	P06733	Alpha-enolase	ENOA_HUMAN	7.01	47481	65	179
16	2694	P00441	Superoxide dismutase [Cu-Zn]	SODC_HUMAN	5.70	16154	64	72
17	518	P18206	Vinculin	VINC_HUMAN	5.50	124292	29	176
18	172	P21333	Filamin-A	FLNA_HUMAN	5.70	283301	10	61
19	548	P12814	Alpha-actinin-1	ACTN1_HUMAN	5.25	103563	28	112
20	2485	P23528	Cofilin-1	COF1_HUMAN	8.22	18719	57	58
21	2823	P05413	Fatty acid-binding protein, heart	FABPH_HUMAN	6.29	14906	67	92
22	110	P21333	Filamin-A	FLNA_HUMAN	5.70	283301	18	88
23	234	P12110	Collagen alpha-2(VI) chain	CO6A2_HUMAN	5.85	109709	14	57
24	224	P18206	Vinculin	VINC_HUMAN	5.50	124292	41	276
25	2687	Q96NR8	Retinol dehydrogenase 12	RDH12_HUMAN	9.73	35528	22	57
26	346	Q9HAE3	EF-hand calciumbinding domaincontaining protein 1	EFCB1_HUMAN	4..91	24757	35	58
27	2319	Q9ULE0	Protein WWC3	WWC3_HUMAN	6.00	123741	13	60
28	341	P11142	Heat shock cognate 71 kDa protein	HSP7C_HUMAN	5.37	71082	50	189
29	307	P12814	Alpha-actinin-1	ACTN1_HUMAN	5.25	103563	37	128
30	1860	P62736	Actin, aortic smooth muscle	ACTA_HUMAN	5.23	42381	42	198
31	603	Q92737	Ras-like protein family member 10A	RSLAA_HUMAN	9.79	22812	32	58
32	1569	Q96S15	GATOR complex protein WDR24	WDR24_HUMAN	6.11	10399	15	59
33	2827	Q9NQ76	Matrix extracellular phosphoglycoprotein	MEPE_HUMAN	8.62	58498	18	57
34	2846	Q5VWT5	FYN-binding protein 2	FYB2_HUMAN	8.58	82703	14	70
35	1114	P00352	Retinal dehydrogenase 1	AL1A1_HUMAN	6.30	55454	44	120
36	2559	Q01995	Transgelin	TAGL_HUMAN	8.87	22653	60	96
37	2928	P06702	Protein S100-A9	S10A9_HUMAN	5.71	13291	65	58
38	1842	P35232	Prohibitin	PHB_HUMAN	5.57	29843	38	73
39	1050	P08670	Vimentin	VIME_HUMAN	5.06	53676	28	69
40	2399	P35228	Nitric oxide synthase, inducible	NOS2_HUMAN	8.20	132573	18	57
41	865	P02545	Prelamin-A/C	LMNA_HUMAN	6.57	74380		240
42	702	P02787	Serotransferrin	TRFE_HUMAN	6.81	79280	38	138
43	2847	Q9BWT1	Cell division cycleassociated protein 7	CDCA7_HUMAN	9.57	43458	28	57
44	1344	P05787	Keratin, type II cytoskeletal 8	K2C8_HUMAN	5.52	53671	47	168
45	1341	P68032	Actin, alpha cardiac muscle 1	ACTC_HUMAN	5.23	42334	51	122
46	571	Q9C0H9	SRC kinase signaling inhibitor 1	SRCN1_HUMAN	9.32	112682	9	61
47	1696	P68032	Actin, alpha cardiac muscle 1	ACTC_HUMAN	5.23	42334	51	123

48	2478	P63267	Actin, gammaenteric smooth muscle	ACTH_HUMAN	5.31	42249	35	65
49	1633	P62937	Peptidyl-prolyl cistrans isomerase A	PPIA_HUMAN	7.68	18229	61	68
50	2442	O95789	Zinc finger MYMtype protein 6	ZMYM6_HUMAN	8.59	151755	16	59
51	239	Q8IYX0	Zinc finger protein 679	ZN679_HUMAN	9.23	48687	22	57
52	1588	P17661	Desmin	DESM_HUMAN	5.21	53560	34	80
53	649	P11142	Heat shock cognate 71 kDa protein	HSP7C_HUMAN	5.37	71081	18	56

[a] *Protein accession number for SWISSPROT Database.*

[b] *Theoretical isoelectric point.* [c] *Theoretical relative mass.* [d] *MASCOT coverage*

[e] *MASCOT score*
